# Incidence and Risk Factors for Postoperative Delirium in Patients Undergoing Spine Surgery: A Systematic Review and Meta-Analysis

**DOI:** 10.1155/2019/2139834

**Published:** 2019-11-26

**Authors:** Xinjie Wu, Wei Sun, Mingsheng Tan

**Affiliations:** ^1^Peking University China–Japan Friendship School of Clinical Medicine, Beijing 100029, China; ^2^Department of Orthopedic Surgery, China-Japan Friendship Hospital, Beijing 100029, China

## Abstract

**Background:**

The present study aims to investigate the incidence and risk factors associated with postoperative delirium in patients undergoing spine surgery.

**Methods:**

PubMed, EMBASE, Cochrane Library, and Science Citation Index were searched up to August 2019 for studies examining postoperative delirium following spine surgery. Incidence and risk factors associated with delirium were extracted. Odds ratios (OR) and 95% confidence intervals (CI) were calculated for outcomes. The Newcastle–Ottawa Scale (NOS) was used for the study quality evaluation.

**Results:**

The final analysis includes a total of 40 studies. The pooled analysis reveals that incidence of delirium is 8%, and there are significant differences for developing delirium in age (OR 1.07; 95% CI 1.04–1.09), age more than 65 (OR 4.77; 95% CI 4.37–5.16), age more than 70 (OR 15.87; 95% CI 6.03–41.73), and age more than 80 (OR 1.91; 95% CI 1.78–2.03) years, male (OR 0.81; 95% CI 0.76–0.86), a history of alcohol abuse (OR 2.11; 95% CI 1.67–2.56), anxiety (OR 1.74; 95% CI 1.04–2.44), congestive heart failure (OR 1.4; 95% CI 1.21–1.6), depression (OR 2.5; 95% CI 1.52–3.49), hypertension (OR 1.12; 95% CI 1.04–1.2), kidney disease (OR 1.41; 95% CI 1.16–1.66), neurological disorder (OR 4.66; 95% CI 4.22–5.11), opioid use (OR 1.86; 95% CI 1.18–2.54), psychoses (OR 2.77; 95% CI 2.29–3.25), pulmonary disease (OR 1.81; 95% CI 1.27–2.35), higher mini-mental state examination (OR 0.7; 95% CI 0.5–0.89), preoperative pain (OR 1.88; 95% CI 1.11–2.64), and postoperative urinary tract infection (OR 5.68; 95% CI 2.41–13.39).

**Conclusions:**

A comprehensive understanding of incidence and risk factors of delirium can improve prevention, diagnosis, and management. Risk of postoperative delirium can be reduced based upon identifiable risk factors.

## 1. Introduction

Postoperative delirium is a common complication after surgery in the elderly and causes difficulty in postoperative care [1, 2]. It is defined as an acute change in the cognitive status characterized by fluctuating consciousness, attention, memory, perceptions, and behavior postoperatively [3]. Postoperative delirium often brings out many adverse outcomes, such as functional disability, increased health care costs, and higher morbidity and mortality rates [4]. Thus, a further understanding and prevention of delirium may help reduce these problems and the associated costs. Some previous studies have reported the incidence and risk factors for delirium. However, incidences of postoperative delirium differ greatly, and risk factors of these studies are inconsistent. Therefore, we perform a systematic review and meta-analysis to explore incidence and risk factors for developing postoperative delirium following spine surgery.

## 2. Materials and Methods

### 2.1. Search Strategy

The systematic review and meta-analysis were done according to the Preferred Reporting Items for Systematic Reviews and Meta-Analyses (PRISMA) guideline and AMSTAR (assessing the methodological quality of systematic reviews) Guidelines [5, 6]. PubMed, EMBASE, Cochrane Library, and Science Citation Index were searched exhaustively with inception to August 2019. The language was restricted to English, and only published articles were included. The search terms were combinations of epidemiology, prevalence, incidence, delirium, deliriums, deliria, delirious, confusion, transient mental disorder, spine, spinal cord, vertebrae, surgery, and operation. Papers from the reference lists of included studies and other meta-analyses were also searched.

### 2.2. Selection Criteria

Studies included in this systematic review and meta-analysis met the following criteria: (1) original articles on patients who underwent spine surgery, (2) observational, case series or cohort study design, (3) at least incidence reported or one risk factor identified as being associated with delirium, and (4) full text available. If the inclusion criteria were not met, the study was excluded. If the same study was published in different years or various journals, then the most frequently cited study was included for this meta-analysis. The potentially qualified studies were selected independently by 2 authors according to the inclusion and exclusion criteria. Any discrepancy was resolved by discussion to reach a consensus.

### 2.3. Data Extraction

Data were extracted by two independent authors. By discussion or by involving a third author, disagreements were addressed. The general features cover the first author, publication year, country, study type, sample size, patient characteristics, patients who underwent surgery, delirium diagnosis tool, incidence duration of delirium, and significant factors.

### 2.4. Quality Assessment

Two authors independently evaluated the quality of the studies, and the level of agreement between them was recorded. Any disagreements between the 2 authors were resolved by discussion with a third author. Newcastle–Ottawa Scale (NOS) was utilized to assess the quality of each study [7] since no studies were randomized controlled trials. Studies with 7–9 points could be identified as high quality, 5–6 points as moderate quality, and 0–4 as poor quality.

### 2.5. Statistical Analysis

The meta-analysis of comparable data was performed using Stata/SE version 15.0 software. All adjusted odds ratio (OR) with 95% confidence interval (CI) were collected and pooled to evaluate the relationships between various risk factors and postoperative delirium in patients undergoing spine surgery. In addition, crude ORs with 95% CIs were calculated based on the frequency reported in the original literature. Inconsistency was quantified with *I*^2^ statistic, and an *I*^2^ of >50% was considered to indicate substantial heterogeneity. The random-effects model or the fixed-effect model was used depending on the heterogeneity of studies included. A random-effects model was used for heterogeneous data. Otherwise, a fixed-effect model was used. Begg's and Egger's test were used to estimate publication bias, when 10 or more studies are presented. For any variable presenting with large heterogeneity, sensitive analysis or subgroup analysis was used to investigate the potential origin of heterogeneity.

### 2.6. Search Results

**Figure 1:**
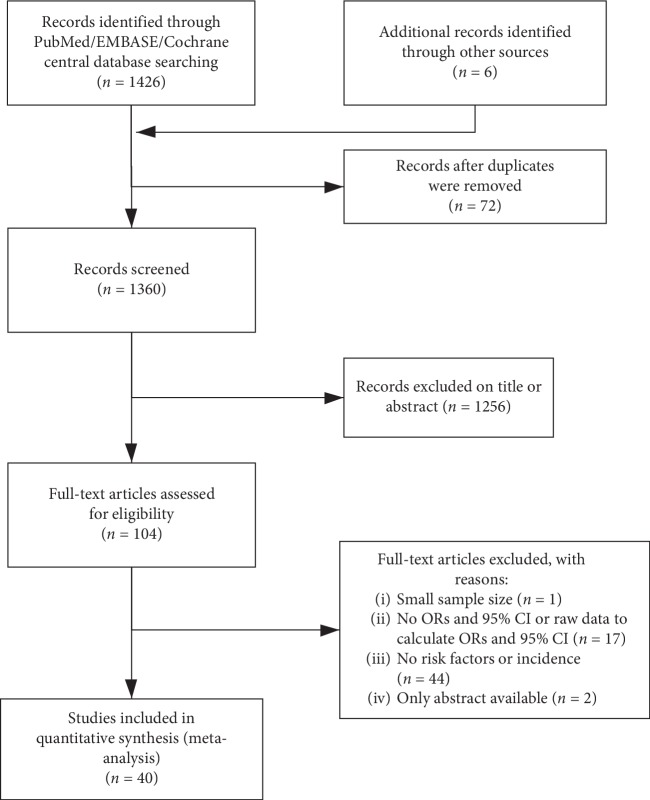
Flow chart of the literature search and article selection.

There were 1360 relevant studies included according to the search strategy. After the titles and abstracts were reviewed, 1256 of them were removed. A full-text review was evaluated in the 104 records maintained, and 64 of them were excluded because they did not meet the inclusion criteria. Finally, 40 studies representing 712820 patients were included in the present meta-analysis (Figure 1).

### 2.7. Study Characteristics and Quality Assessment

The characteristics of the included studies are summarized in Table 1. 22 studies were conducted in Asian countries, 16 studies in North America, and 2 studies in Europe. 31 studies were retrospective, and 9 were prospective in design. The sample size ranged from 35 to 578457 patients. The reported incidence of delirium ranged from 0.49% to 31.43% for patients after spinal surgery. To evaluate the quality of each study, the NOS was utilized. In those studies, all of them were of moderate to high quality (range, 6–8) (Table 1).

### 2.8. Incidence of Postoperative Delirium after Spine Surgery

**Figure 2:**
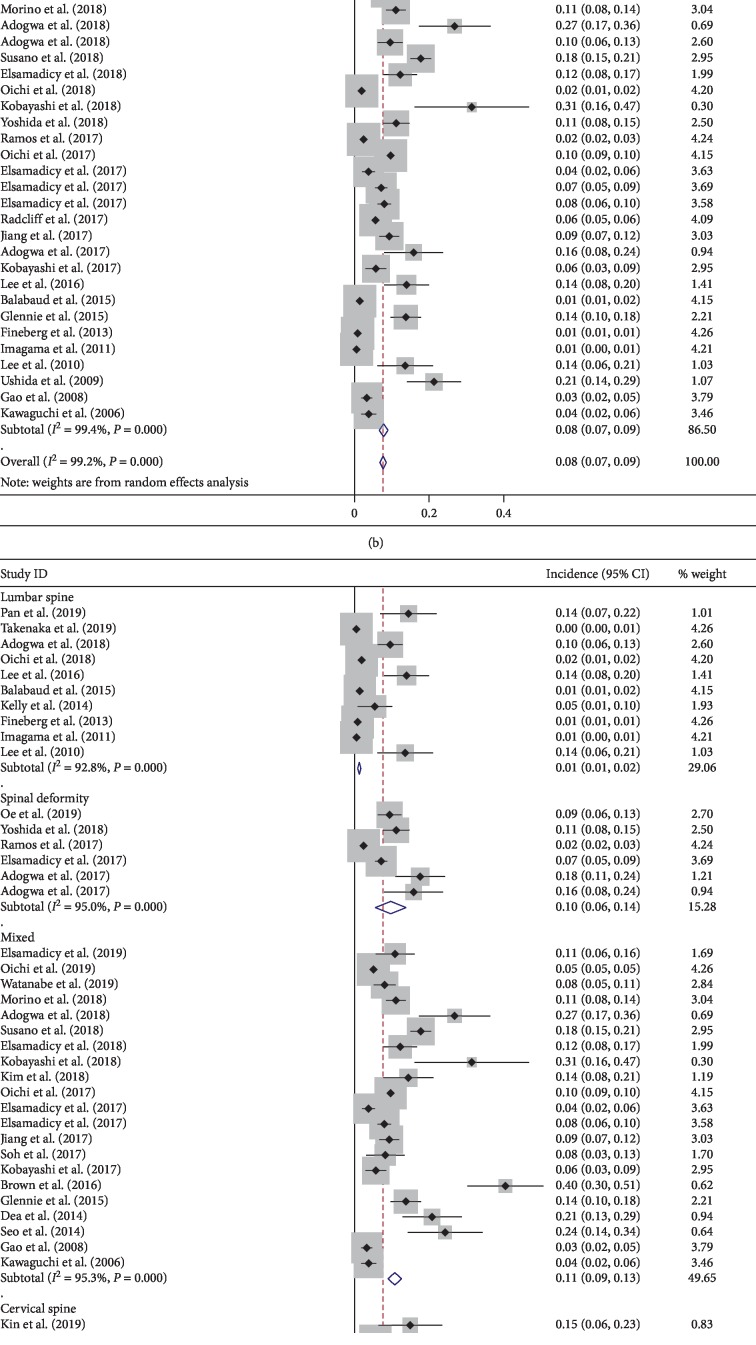
Pooled result of incidence of delirium: (a) subgroup analysis based on the factor of country; (b) subgroup analysis based on the factor of study type; (c) subgroup analysis based on the factor of surgical site; (d) result of sensitive analysis; (e) Begg's funnel plot.

The final meta-analysis included 40 studies [1, 8–46] from 7 different countries, and the pooled incidence was 8% (Figure 2). There was high heterogeneity (*I*-squared > 50%, *P* < 0.001). Interestingly, the heterogeneity remained high with each of the subgroups of study type, countries, or operated levels (Figure 2(a)–2(c)). After sensitive analysis, 3 studies [11, 25, 41] showed great influence on the pooled result (Figure 2(d)). The asymmetry Begg's funnel plot suggested the presence of publication bias for incidence of postoperative delirium after spine surgery (*P* < 0.001) (Figure 2(e)).

### 2.9. Risk Factors for Postoperative Delirium after Spine Surgery

The ORs and 95% CIs of the risk factors are displayed in Table 2. Among these, 33 factors were examined in 2 or more studies and 18 factors demonstrated statistical significance.

After synthesis of 7 studies, it revealed that patients who developed delirium were significantly older (OR 1.07; 95% CI 1.04–1.09). Meanwhile, age older than 65 (OR 4.77; 95% CI 4.37–5.16), 70 (OR 15.87; 95% CI 6.03–41.73), and 80 (OR 1.91; 95% CI 1.78–2.03) years were significantly associated with the risk of developing delirium. Another demographic factor male was considered to be associated with less delirium risk in the pooled analysis (OR 0.81; 95% CI 0.76–0.86).

A history of alcohol abuse (OR 2.11; 95% CI 1.67–2.56), anxiety (OR 1.74; 95% CI 1.04–2.44), congestive heart failure (OR 1.4; 95% CI 1.21–1.6), depression (OR 2.5; 95% CI 1.52–3.49), hypertension (OR 1.12; 95% CI 1.04–1.2), kidney disease (OR 1.41; 95% CI 1.16–1.66), neurological disorder (OR 4.66; 95% CI 4.22–5.11), opioid use (OR 1.86; 95% CI 1.18–2.54), psychoses (OR 2.77; 95% CI 2.29–3.25), and pulmonary disease (OR 1.81; 95% CI 1.27–2.35) were more likely to develop delirium than controls. Assessment of mental state, as measured by mini-mental state examination (MMSE), demonstrated a significantly lower risk to develop delirium in patients with higher scores (OR 0.7; 95% CI 0.5–0.89). In addition, preoperative pain and postoperative urinary tract infection (UTI) were related to the development of delirium (OR 1.88; 95% CI 1.11–2.64 and OR 5.68; 95% CI 2.41–13.39, respectively).

## 3. Discussion

Delirium is thought to be a less transient disorder than previously believed in several studies [8, 11]. In addition, it has been reported that patients with postoperative delirium have a higher mortality rate than in those without it [4]. Due to the fact that delirium is varying and multifactorial, it will be helpful for prevention of delirium through identifying predictable risk factors.

This systematic review and meta-analysis were performed to pool and identify the incidence and risk factors of postoperative delirium after spine surgery. The pooled incidence of delirium in this meta-analysis is 8%. However, the present study showed wide variation and heterogeneity in incidence of delirium. A previous meta-analysis of 6 studies reported incidence of delirium after spine surgery varies from 0.84% to 21.3% [47]. Interestingly, the heterogeneity remained high with each of the subgroups of study type, countries, or operated levels (Figures 2(a)–2(c)). We found that patients with spinal deformity have higher rate of delirium (10%) and lower rate in patients with lumbar spine (1%). Meanwhile, prospective studies have a higher incidence of postoperative delirium than retrospective studies. After sensitive analysis, 3 studies [11, 25, 41] showed great influence on the pooled result (Figure 2(d)). All these 3 studies have relatively a larger sample size (range, 13188 to 578457), low incidence of delirium (range, 0.49 to 5.1%), and retrospective nature of study design, which may contribute to the heterogeneity. The asymmetry Begg's funnel plot suggested the presence of publication bias for incidence of postoperative delirium after spine surgery, and lower incidence values could be missing (Figure 2(e)).

**Figure 3:**
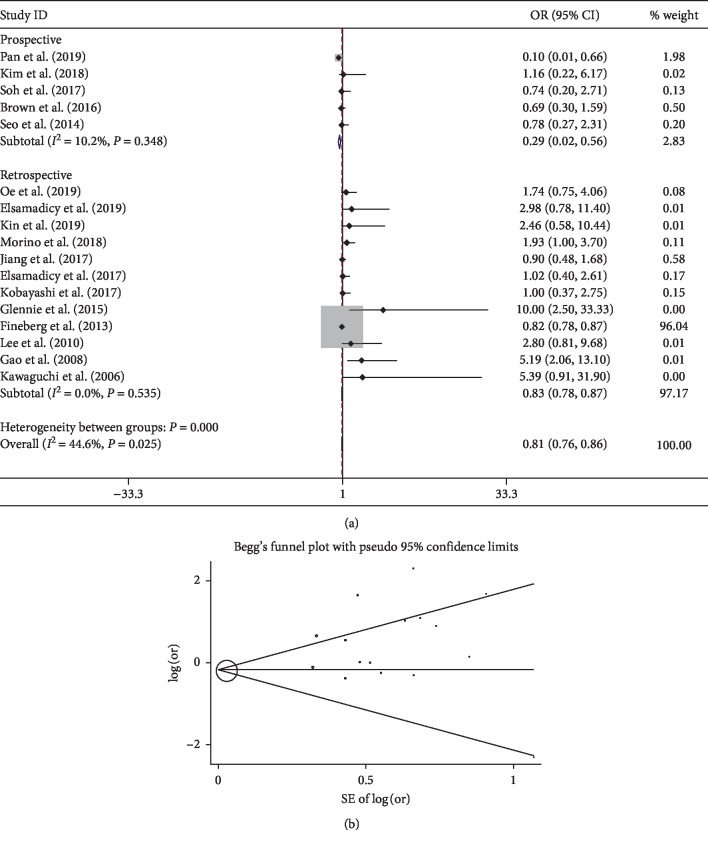
Pooled result of male: (a) subgroup analysis based on the factor of study type; (b) Begg's funnel plot.

One of the most important risk factors was older age, especially in patients over 65. This may be attributed to the fact that elderly patients are more likely influenced by age-related physical and psychical changes. Aging is also associated with a higher incidence of comorbidity such as hypertension, diabetes mellitus, and pulmonary disease [12, 30]. The highest rate of delirium in our meta-analysis is 31.43% in a multicenter prospective study with patient's age more than 90 [21]. Another significant demographic factor is male as a protective factor. Through subgroup analysis, we found that study design may contribute to the heterogeneity and prospective studies showing relatively a higher risk of developing delirium in females (Figure 3(a)). For publication bias, Begg' funnel plot demonstrated no significant bias (Figure 3(b)).

**Figure 4:**
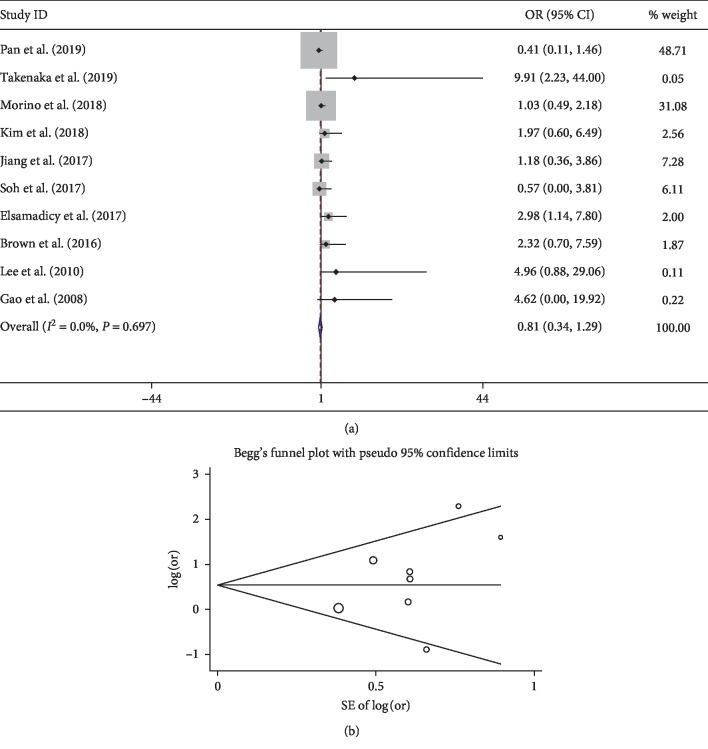
Pooled result of cardiovascular comorbidity: (a) forest plot of cardiovascular comorbidity; (b) Begg's funnel plot.

The present study showed that comorbidities significantly increase the risk of postoperative delirium after spine surgery. A history of alcohol abuse, congestive heart failure, hypertension, neurological disorder, opioid use, psychoses, and pulmonary disease are related to develop delirium. However, diabetes mellitus, history of surgery, and cerebral vascular diseases were not found to be related to developing delirium, which was consistent with the previous meta-analysis [47]. For the cardiovascular comorbidity, the pooled result of 10 studies [8, 11, 15, 23, 26, 30, 31, 34, 43, 45] showed no significance (OR 0.81; 95% CI 0.34–1.29) with low heterogeneity (*I*^2^ 0%) (Figure 4(a)). Only one study found cardiovascular comorbidity as a risk factor for delirium [11]. The symmetry Begg's funnel plot suggested no presence of publication bias for cardiovascular comorbidity (Figure 4(b)). Interestingly, however, pooled results showed congestive heart failure as a significant factor. This may be due to the severity of heart diseases.

**Figure 5:**
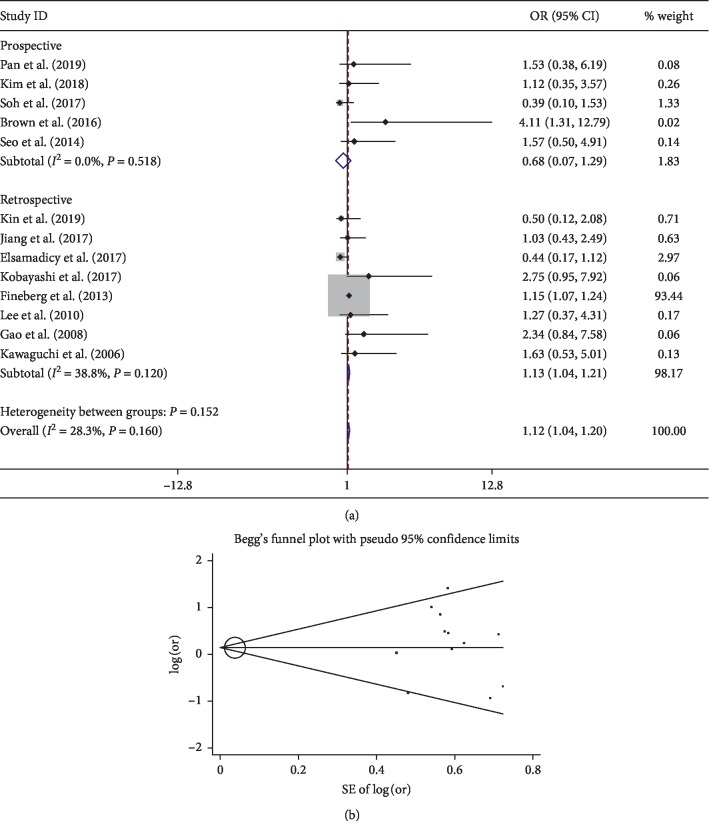
Pooled result of hypertension: (a) forest plot of hypertension; (b) Begg's funnel plot.

Regarding the comorbidity of hypertension, the meta-analysis of 13 studies [1, 8, 12, 23, 26, 30, 31, 34, 39, 41, 43, 45, 46] identified it as a significant factor, and subgroup analysis showed heterogeneity comes from study design (Figure 5(a)). For publication bias, Begg's funnel plot suggested no significant bias (Figure 5(b)). Previous study showed that hypertension leading to microembolization phenomena and cerebral ischemia may be responsible for the occurrence of delirium [48].

**Figure 6:**
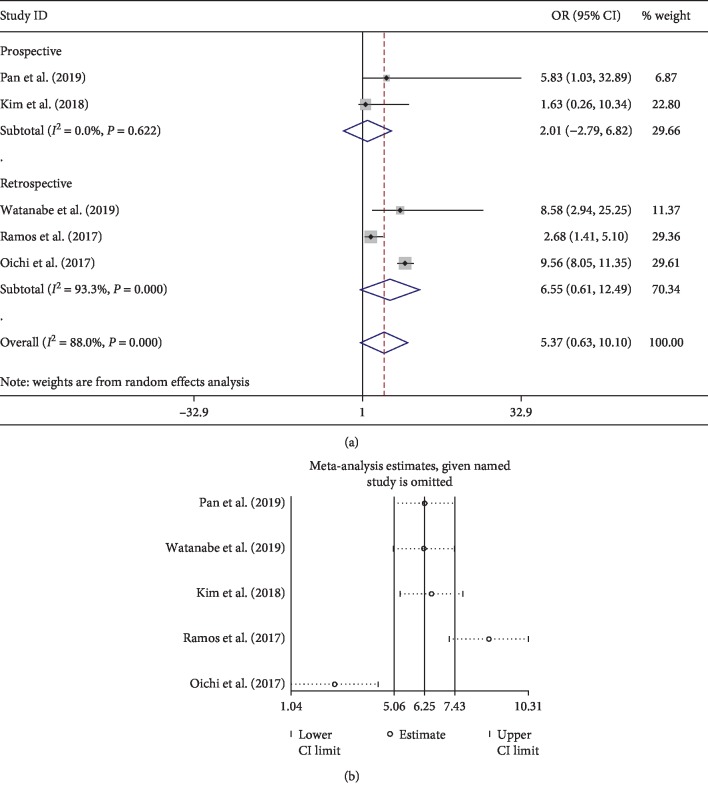
Pooled result of parkinsonism: (a) forest plot of parkinsonism; (b) result of sensitive analysis.

For neurological or mental diseases, neurological disorder, psychoses, anxiety, and depression were found to be associated with developing delirium. The meta-analysis of 5 studies showed that mild cognitive impairment is not related to the occurrence of delirium (OR 2.43; 95% CI 0.99–3.86; *I*^2^ 0%). Meanwhile, parkinsonism was also not found to be related to postoperative delirium (OR 5.37; 95% CI 0.63–10.1). However, there is still controversy in the role of parkinsonism for postoperative delirium. Kim et al. [23] found that that parkinsonism is not a risk factor for postoperative delirium after multivariable analysis. Interestingly, Pan et al. [8] found an opposite result, which may be attributed to relatively a smaller sample of patients with parkinsonism in their study. Notably, the result should be explained with caution since the heterogeneity is high (*I*^2^ 88%). After subgroup analysis, there was a high heterogeneity between retrospective studies (Figure 6(a)). Moreover, the result of sensitive analysis showed two studies [24, 25] contributing greatly to the high heterogeneity (Figure 6(b)). Both studies were retrospective design and focus on patients with parkinsonism, which may result in high heterogeneity.

**Figure 7:**
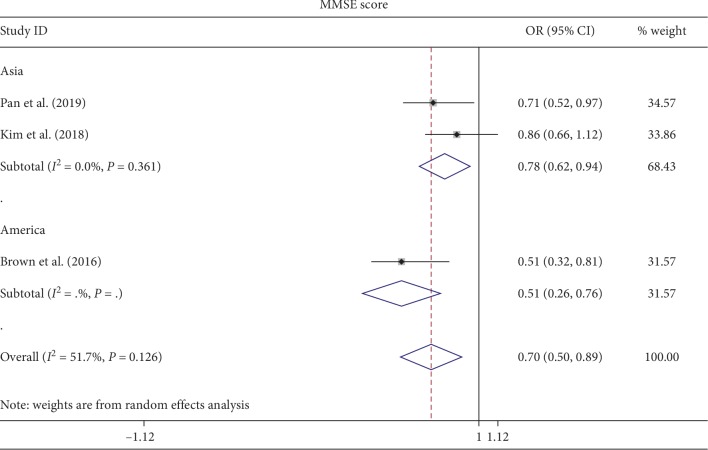
Pooled result of MMSE score. Subgroup analysis based on the factor of country.

Mental states, as assessed by MMSE, were associated with the development of delirium (OR 0.7; 95% CI 0.5–0.89). Through subgroup analysis, we found that geographical factors may contribute to heterogeneity (Figure 7). This measure of the state of mental health appears to have a clearer association with postoperative delirium compared to Charlson Comorbidity Index (CCI) which assesses the number of specific medical comorbidities. These findings are also seen in other studies where CCI appears less clearly associated with the incidence of delirium in older patients [12, 49].

The finding that preoperative pain and opioid use is associated with increased probability of delirium has been previously reported in patients with or without hip fracture or patients with cancer [49, 50]. In addition, elderly patients are more sensitive to opioid-related adverse events [51]. In patients with spine disease, pain may lead to stress reaction and changes of nerve conduction if not effectively controlled [34]. However, the accumulation of active metabolites in patients receiving opioid may contribute to the psychotic features such as delirium [52]. Hence, it is suggested that a less toxic drug, buprenorphine patch other than morphine, should be considered for patients with osteoarthrosis and other types of lumbago when pain continues despite adequate administrations of nonopioid analgesics [53].

**Figure 8:**
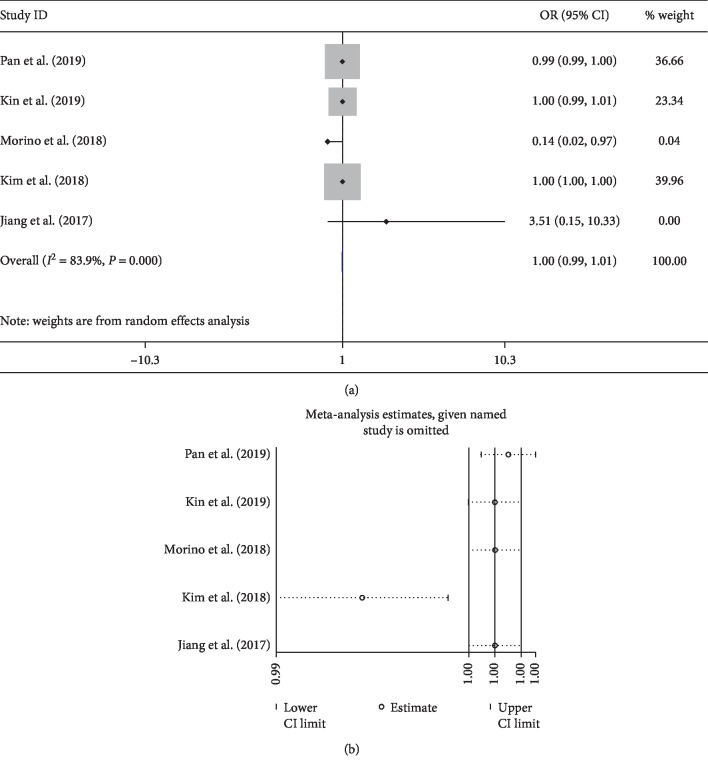
Pooled result of blood loss: (a) forest plot of blood loss; (b) result of sensitive analysis.

**Figure 9:**
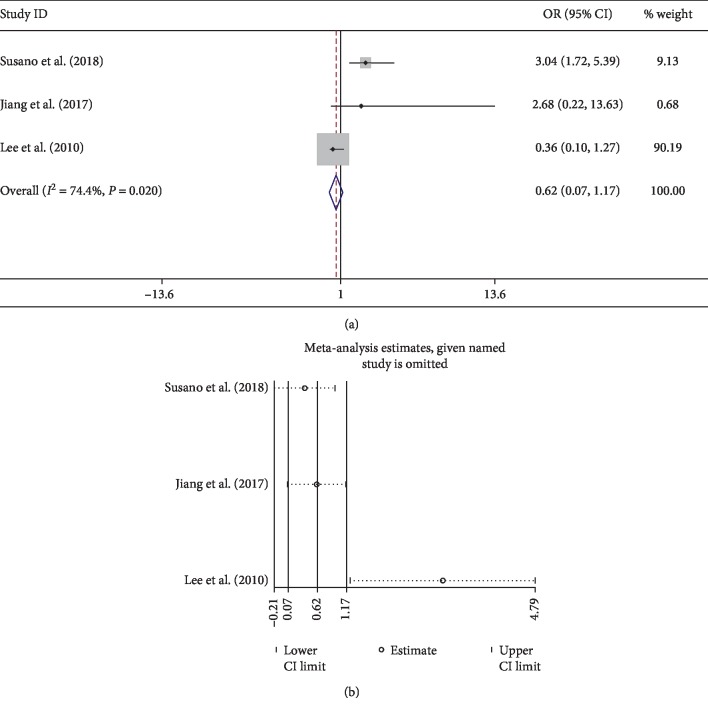
Pooled result of blood transfusion: (a) forest plot of blood transfusion; (b) result of sensitive analysis.

In our study, intraoperative factors do not appear to influence the prevalence of delirium based on normal clinical practice such as blood loss, blood transfusion, cervical surgery, dural tear, operated levels, and operation time. Notably, for intraoperative blood loss, there was high heterogeneity among studies (Figure 8(a)). After sensitive analysis, we found that one study [23] focused on patients with parkinsonism lead to the high heterogeneity. In addition, high heterogeneity was also seen in the meta-analysis of blood transfusion (Figure 9(a)). The sensitive analysis showed that the heterogeneity comes from one study [43], which had more fusion levels (2.27 ± 1.34) and blood loss (1263 ± 903) than other studies (Figure 9(b)). Postoperatively, patients experiencing complications such as UTI had a higher probability to develop delirium.

There are some limitations in our study. First, no randomized controlled trials were included despite our exhausted search from literatures, which may influence the quality of the result. Second, although subgroup analyses were used, the pooled result of incidence was still reported with high heterogeneity, which should be explained with caution.

## 4. Conclusions

In summary, the study reveals that pooled incidence of delirium is 8% and age, gender, history of alcohol abuse, anxiety, congestive heart failure, depression, hypertension, kidney disease, neurological disorder, opioid use, psychoses, pulmonary disease, MMSE, preoperative pain, and postoperative UTI were significant factors for delirium after spine surgery. A comprehensive understanding of incidence and risk factors of delirium can improve prevention, diagnosis, and management.

## Figures and Tables

**Table 1:** Study characteristics and quality assessment.

Author	Publication year	Country	Study type	Sample size	Age mean (SD, range) years	Sex ratio (M : F)	Patients who underwent surgery	Delirium diagnosis tool	Delirium incidence	Duration of delirium (days)	Significant factors	Study quality
Pan et al. [8]	2019	Korea	Prospective	83	71.4 ± 4.6	27 : 56	Lumbar spine	CAM	12/83 (14.5%)	2.6 (1–5)	Male, parkinsonism, lower baseline MMSE score	8
Oe et al. [9]	2019	Japan	Retrospective	319	>18	85 : 234	Spinal deformity	—	30/319 (9.4%)	—	Age, PNI	7
Elsamadicy et al. [10]	2019	USA	Retrospective	138	≥18	40 : 98	Complex spinal fusion (≥5 levels)	—	15/138 (10.9%)	—	Age, intraoperative ketamine use	7
Takenaka et al. [11]	2019	Japan	Prospective	13188	11–94	7174 : 6014	Lumbar spine	—	65/13188 (0.49%)	—	CVD, dural tear	8
Kin et al. [12]	2019	Japan	Retrospective	67	69.6 ± 12.0	49 : 18	Cervical spine	CAM, DSM-IV	10/67 (14.9%)	<3	Low general health perception	8
Oichi et al. [13]	2019	Japan	Retrospective	88370	≥65	47408 : 40962	Lumbosacral, thoracic, cervical, unspecified	—	4502/88370 (5.1%)	—	Age >80	8
Watanabe et al. [14]	2019	Japan	Retrospective	322	75.7 years (67–89)	69 : 253	Thoracic, lumbar spine	—	26/322 (8.1%)	—	Parkinsonism	7
Morino et al. [15]	2018	Japan	Retrospective	532	64.2 (10–89)	283 : 249	Spine	DSM-IV	59/532 (11.1%)	<5	Blood loss	8
Adogwa et al. [16]	2018	USA	Retrospective	82	≥65	33 : 49	Thoracolumbar deformity	CAM	22/82 (18%)	—	Cognitive impairment	7
Adogwa et al. [17]	2018	USA	Retrospective	293	≥18	105 : 188	Lumbar spine	—	28/293 (9.6%)	—	CKD	7
Susano et al. [18]	2018	Portugal	Retrospective	715	73.6 ± 6.0	351 : 400	Cervical, lumbar spine	—	127/715 (17.8%)	—	Age, ASA physical status ≥3, METs <4, depression, nonelective surgery, invasiveness tier 3 or 4, BIS monitoring, mean pain score postoperative day 1	7
Elsamadicy et al. [19]	2018	USA	Retrospective	204	≥60	204 : 0	Elective complex spinal fusion (≥3 levels)	—	25/204 (12.3%)	—	Preoperative hgb level <13.5 g/dl	7
Oichi et al. [20]	2018	Japan	Retrospective	2712	≥20	1738 : 974	Lumbar spine	—	52/2712 (1.9%)	—	Open laminectomy	7
Kobayashi et al. [21]	2018	Japan	Retrospective	35	91.3 (90–98)	14 : 21	Cervical, thoracic, lumbar spine	—	11/35 (31.43%)	—	—	6
Yoshida et al. [22]	2018	Japan	Retrospective	304	62.9 (18–84)	64 : 240	Spinal deformity	—	34/304 (11.2%)	—	Age, operative time ≥6 hours	8
Kim et al. [23]	2018	Korea	Prospective	104	71.7 ± 4.7	36 : 68	Cervical, thoracic, lumbar spine	CAM	15/104 (14.4%)	—	Hyposmia (CCSIT score <9) and RBD (RBDSQ-K >4)	8
Ramos et al. [24]	2017	USA	Retrospective	11043	67 ± 9	2981 : 8062	Spinal deformity	—	269/11043 (2.4%)	—	Movement disorder, parkinsonism	7
Oichi et al. [25]	2017	Japan	Retrospective	6921	≥20	3324 : 3597	Lumbosacral, thoracic, cervical, unspecified	—	670/6921 (9.7%)	—	Parkinsonism	7
Elsamadicy et al. [26]	2017	USA	Retrospective	453	≥65	211 : 242	Spine	DSM-V	17/453 (3.75%)	—	Superficial surgical site infection, UTI, length of hospital stay	8
Elsamadicy et al. [27]	2017	USA	Retrospective	923	≥18	333 : 590	Spinal deformity	—	66/923 (7.15%)	—	Depression, age, operative time, postoperative UTI	7
Elsamadicy et al. [28]	2017	USA	Retrospective	839	≥18	329 : 510	Elective complex spinal fusion (≥3 levels)	—	67/839 (7.98%)	—	—	6
Radcliff et al. [29]	2017	USA	Retrospective	2792	≥65	1487 : 1305	Cervical spine	—	157/2792 (5.6%)	—	Dementia, TIA/stroke, age≥ 85 in cervical decompression patients	7
Jiang et al. [30]	2017	China	Retrospective	451	65.1 (45–84)	226 : 225	Cervical and lumbar spine	—	42/451 (9.3%)	6.7 (4–10)	Intraoperative hypotension <80 mmHg, intraoperative use of dezocine	7
Soh et al. [31]	2017	Korea	Prospective	109	>70	56 : 53	Cervical, thoracic, lumbar spine	ICDSC; CAM-ICU	9/109 (8.2%)	—	Pulmonary disease	7
Adogwa et al. [32]	2017	USA	Prospective e	125	≥65	50 : 75	Spinal deformity	—	22/125 (17.6)	—	—	7
Adogwa et al. [33]	2017	USA	Retrospective	82	≥65	33 : 49	Spinal deformity	—	13/82 (15.9%)	—	Cognitive impairment	7
Kobayashi et al. [1]	2017	Japan	Retrospective	262	82.7 (80–91)	142 : 140	Cervical, thoracic, and lumbar spine	—	15/262 (5.72)	<3	Cervical lesion surgery, blood loss>300 mL	7
Brown et al. [34]	2016	USA	Prospective	195	74 (72–78)	47 : 42	Cervical, lumbar spine	CAM; CAM-ICU; DRS-98-R	36/195 (18.5%)	—	Lower baseline MMSE score, higher average baseline pain, more intravenous fluid, baseline, antidepressant medication	7
Lee et al. [35]	2016	Korea	Retrospective	129	73.5 (70 to 85)	51 : 78	Lumbar spine	CAM; DSM-IV	18/129 (13.9%)	13.2 (1 to 92)	Cognitive impairment	7
Balabaud et al. [36]	2015	France	Retrospective	121	83.2 ± 2.4	48 : 73	Lumbar spine	—	16/(13%)	—	Instrumentation, blood loss	7
Glennie et al. [37]	2015	Canada	Retrospective	276	42.9 ± 18.8	190 : 86	Thoracic, lumbar spine	—	38/276 (13.8%)	—	Age, male, head injury	7
Dea et al. [38]	2014	Canada	Prospective	101	62 (33–85)	50 : 51	Thoracic, lumbar, sacral spine	—	21/101 (20.8)	—	—	6
Seo et al. [39]	2014	Korea	Prospective	70	70.1 ± 5.8	32 : 38	Cervical, lumbar spine	ICDSC; CAM-ICU	17/70 (24.3%)	—	Preoperative GDS, BIS measured intraoperatively under 40	8
Kelly et al. [40]	2014	Canada	Prospective	92	66.08 ± 10.59	—	Lumbar spine	—	5/92 (5.4%)	—	CCI, dural tear	8
Fineberg et al. [41]	2013	USA	Retrospective	578457	>18	285520 : 292937	Lumbar spine	—	4857/578457 (0.84%)	—	Age ≥65, teaching hospital, alcohol abuse, deficiency anemia, congestive heart failure, coagulopathy, depression, DM with end-organ damage, drug abuse, hypertension, fluid/electrolyte disorders, metastatic neoplasm, neurological disorder, psychoses, pulmonary circulation disorders, renal failure, weight loss	6
Imagama et al. [42]	2011	Japan	Retrospective	918	54 (11–87)	521 : 397	Lumbar spine	—	5/918 (0.54%)	—	—	6
Lee et al. [43]	2010	Korea	Retrospective	87	73.5 (70–85)	27 : 50	Lumbar spine	CAM, DSM-IV	11/81 (13.6%)	13.2 (1 to 92)	Cerebral vascular disease, low hemoglobin and hematocrit levels at 1 day after surgery, bad nutritional status	7
Ushida et al. [44]	2009	Japan	Retrospective	122	52–86	—	Cervical spine	DOS, DSM-IV	26/122 (21.3%)	—	Age >70, high-dose methylprednisolone (>1000 mg), hearing impairment	6
Gao et al. [45]	2008	China	Retrospective	549	48.2 (10–83)	302 : 247	Cervical, thoracic, lumbar, sacral spine	DOS, DSM-IV	18/549 (3.3%)	3.1 (1 to 8)	Central nervous system disorder, surgical history, age> 65, DM, blood transfusion ≥800 ml, hemoglobin <100 g/L	7
Kawaguchi et al. [46]	2006	Japan	Retrospective	341	59.2 (14–88)	186 : 155	Cervical, thoracic, lumbar, sacral spine	CAM, DSM–III–R	13/341 (3.8%)	≤7	Low concentrations of hemoglobin and hematocrit 1 day after surgery, ambulatory status at admission	8

DOS, delirium observation screening scale; DSM, diagnostic and statistical manual of mental disorders; CAM, confusion assessment method; MMSE, mini-mental state examination; CVD, cardiovascular disease; CKD, chronic kidney disease; CAM-ICU, confusion assessment method for the intensive care unit; DRS-98-R, delirium rating scale revised-98; ICDSC, intensive care delirium screening checklist; PNI, Prognostic Nutritional Index; ASA, American Society of Anesthesiologists physical status; BIS, Bispectral Index; METs, metabolic equivalents of task; UTI, urinary tract infection; RBD, rapid eye movement sleep behavior disorder; CCSIT, cross-cultural smell identification test; RBDSQ-K, Korean version of RBD screening questionnaire; TIA, transient ischemic attack; GDS, global deterioration scale; BIS, Bispectral Index; CCI, Charlson Comorbidity Index.

**Table 2:** Outcomes of meta-analysis for risk factors.

Risk factors	No. of studies	Pooled OR (95% CI)	Heterogeneity *I*^2^ (%)	*P* value	Effects model
Admission to ICU	3	2.51 (0.38–4.64)	0	0.944	Fixed
Age	7	1.07 (1.04–1.09)	16.5	0.304	Fixed
Age >65	3	4.77 (4.37–5.16)	0	0.383	Fixed
Age >70	3	15.87 (6.03–41.73)	48	0.14	Fixed
Age >80	2	1.91 (1.78–2.03)	0	0.844	Fixed
Alcohol abuse	4	2.11 (1.67–2.56)	0	0.397	Fixed
Anxiety	2	1.74 (1.04–2.44)	0	0.773	Fixed
Blood loss	5	1 (0.99–1.01)	83.9	<0.001	Random
Blood transfusion	3	0.62 (0.07–1.17)	74.4	0.02	Random
Cardiovascular comorbidity	10	0.81 (0.34–1.29)	0	0.697	Fixed
CCI	2	1.26 (0.56–1.96)	0	0.355	Fixed
Cervical surgery	6	0.97 (0.45–1.48)	0	0.514	Fixed
Congestive heart failure	3	1.4 (1.21–1.6)	0	0.708	Fixed
Depression	7	2.5 (1.52–3.49)	76	<0.001	Random
DM	13	1.09 (0.6–1.59)	0	0.978	Fixed
Dural tear	2	3.21 (0.07–6.35)	0	0.864	Fixed
Gender (male)	17	0.81 (0.76–0.86)	44.6	0.025	Fixed
History of surgery	6	1.09 (0.55–1.64)	0	0.617	Fixed
Hypertension	13	1.12 (1.04–1.2)	28.3	0.16	Fixed
Kidney disease	6	1.41 (1.16–1.66)	0	0.92	Fixed
MMSE score	3	0.7 (0.5–0.89)	51.7	0.126	Random
Neurological disorder	4	4.66 (4.22–5.11)	0	0.521	Fixed
Operated levels	2	1.02 (0.81–1.22)	0	0.523	Fixed
Operation time	4	1 (0.99–1)	0	0.725	Fixed
Parkinsonism	5	5.37 (0.63–10.1)	88	<0.001	Random
Preoperative VAS	2	1.88 (1.11–2.64)	0	0.816	Fixed
Previous cerebral vascular diseases	7	1.82 (0.7–2.94)	0	0.952	Fixed
Previous mild cognitive impairment	5	2.43 (0.99–3.86)	0	0.967	Fixed
Previous opioid use	3	1.86 (1.18–2.54)	0	0.659	Fixed
Psychoses	5	2.77 (2.29–3.25)	0	0.474	Fixed
Pulmonary disease	6	1.81 (1.27–2.35)	0	0.925	Fixed
Postoperative UTI	2	5.68 (2.41–13.39)	0	0.463	Fixed
Superficial surgical site infection	2	0.28 (-3.25-3.81)	0	0.433	Fixed

CCI, Charlson Comorbidity Index; DM, diabetes mellitus; MMSE, mini-mental state examination; VAS, Visual Analogue Scale; UTI, urinary tract infection.
